# Laryngeal allergy

**DOI:** 10.20407/fmj.2020-022

**Published:** 2020-11-13

**Authors:** Kensei Naito, Hisayuki Kato, Yuki Inuzuka, Ichiro Tateya

**Affiliations:** 1 Academy of Nursing, Fujita Health University, Toyoake, Aichi, Japan; 2 Department of Otolaryngology - Head and Neck Surgery, Fujita Health University, School of Medicine, Toyoake, Aichi, Japan

**Keywords:** Laryngeal allergy, Chronic cough, Atopic cough, Cough variant asthma, Gastroesophageal reflux disease

## Abstract

Many patients with allergic rhinitis have accompanying laryngeal symptoms such as persistent cough and/or globus. Chronic laryngeal allergy is suspected to be an important cause of these laryngeal symptoms. We have been working toward establishing the concept of a new pathological condition termed “laryngeal allergy” since 1988. In Japan, the first diagnostic criteria for laryngeal allergy were established in 1995. However, these early criteria were inadequate because there was inadequate distinction between laryngeal allergy and other causes of persistent cough and globus. Therefore, more advanced criteria were reconstructed from a completely different viewpoint in 2005 to correctly distinguish laryngeal allergy from other similar diseases. The criteria established in 2005 were modified slightly in 2011 to improve the diagnostic accuracy based on the results of fundamental and clinical investigations. The Japanese Respiratory Society (JRS) included chronic laryngeal allergy in the diagnostic flowchart of the JRS guidelines for the management of cough and sputum in 2019, and chronic laryngeal allergy has recently gained wider recognition in Japan. The accurate diagnosis of conditions resembling laryngeal allergy is important in controlling cough and/or globus and preventing the unnecessary use of medical resources. Therefore, further investigations are warranted to better understand laryngeal allergy and similar diseases.

## Introduction

Since 1988, we have been working toward establishing the concept of a new pathological condition termed “laryngeal allergy”, as many patients with allergic rhinitis have accompanying laryngeal symptoms such as persistent cough and/or globus that are suspected to be cause by laryngeal allergy.^1^ Several researchers from the Department of Otolaryngology in Fujita Health University and other institutes voluntarily formed an inquiry group to elucidate the existence of laryngeal allergy in 1988, as no systematic and methodological investigations on laryngeal allergy had previously been conducted in Japan. Subsequently, many studies on laryngeal allergy have been successfully conducted.^1–5^ In 2011, the inquiry group was assigned to the Japan Laryngological Association (JLA), a member of the Japanese Official Academic Societies, as the formal committee for the standardization of diagnostic criteria for laryngeal allergy. Several differential diagnoses for laryngeal allergy have been proposed in recent decades. The Japanese Respiratory Society (JRS) published the JRS guidelines for the management of cough and sputum in 2019,^6^ which recognized laryngeal allergy as a cause of persistent cough. Laryngeal allergy is now generally recognized in Japan. To acknowledge their achievements, the prominent research of the committee members related to laryngeal allergy is presented in this review.

## History

Williams (1972)^7^ and Pang (1974)^8^ published early summaries of the history of laryngeal allergy. At the early stage in the history of laryngeal allergy, laryngeal allergy was confused with hereditary angioneurotic edema caused by a congenital deficiency of C1 inhibitor, which is the primary inhibitor of the complement pathway.^9^ The first presentation of laryngeal allergy was reported in 1940, and was a case of angioneurotic edema of the larynx due to sensitivity to chicle, which is a raw material used to make chewing gum.^10^ Subsequently, there have been several case reports of laryngeal allergy due to antibiotics,^11^ bee sting,^12^ serum injection,^13^ and mosquito bite.^14^ A study published in 1966 reported that 42 of 48 patients with spasmodic croup and laryngotracheitis developed laryngeal allergy, and stressed the association between allergy and upper airway inflammation.^15^ In 1968, Alinov^16^ reported the causes of allergies in 69 of 245 patients with laryngitis, and administered antigen-specific immunotherapy to this patient group. Williams (1972)^7^ reported that the antigens in 22 patients with laryngeal allergy included dust, mold, wheat, corn, egg, milk, beef, chocolate, tomato, penicillin, and iodine; this study mentioned that laryngeal allergy had been ignored as a causative factor because its existence had been poorly recognized.

In 1974, Pang^8^ categorized laryngeal allergy into acute and chronic types. Acute or anaphylactic laryngeal allergy causes rapid and fatal laryngeal stenosis, and an ICD-10 code was assigned for this patient group who require occasional hospitalization to receive immediate treatment or a series of treatments. In contrast, it has proven difficult to accurately diagnose chronic laryngeal allergy, which resembles allergic rhinitis or bronchial asthma (BA). Chronic laryngeal allergy was initially assumed to involve simple chronic inflammation of the larynx,^8^ and efforts to define chronic laryngeal allergy subsequently declined in Western countries.

In Japan, volunteer researchers founded an inquiry group in 1988 to study chronic laryngeal allergy, as no large-scale studies had investigated laryngeal allergy. The group members published studies on chronic laryngeal allergy,^2,4,5^ and the first diagnostic criteria for chronic laryngeal allergy were established in 1995. Although these initial criteria were unable to distinguish chronic laryngeal allergy from other diseases that cause persistent cough and globus, more advanced criteria were reconstructed from a completely different viewpoint in 2005 to correctly distinguish laryngeal allergy from similar diseases, such as BA, cough variant asthma (CVA), eosinophilic bronchitis (EB), atopic cough (AC), gastroesophageal reflux disease (GERD), postnasal drip syndrome (PNDS), post-infectious cough (PIC), radiotransparent foreign body in the respiratory tract, and psychogenic cough.^17^ In 2011, the inquiry group was designated by the JLA as the formal committee for the standardization of criteria for the diagnosis of chronic laryngeal allergy. A secretariat of the committee was established in the Department of Otolaryngology, Fujita Health University, to support each investigation and the joint research projects performed by the committee members. The criteria established in 2005 were slightly modified in 2011 to improve the diagnostic accuracy.^17^ The JRS published guidelines for the management of cough and sputum in 2019,^6^ which included chronic laryngeal allergy in the diagnostic flowchart. In recent years, chronic laryngeal allergy has gained wider recognition in Japan.

## Fundamental and clinical investigations

In Japan, the prevalence of cedar pollinosis has increased in recent decades because of the increase in the amount of pollen and changes in social environments.^18,19^ Several clinicians have reported treating patients with cedar pollinosis with accompanying laryngeal symptoms, and this condition was suspected to be caused by laryngeal allergy.^1^ The notion that laryngeal manifestations in patients with cedar pollinosis might be generated by an allergic reaction of the larynx is supported by fundamental studies involving animal sensitization experiments.^1,20^ Ishida et al.^21^ demonstrated that mucosal mast cells, which are major allergic inflammatory cells, primarily accumulate in the arytenoid and subglottic epithelium. Furthermore, microfold cells (antigen-sampling cells overlying the lymphoid follicles in the gastrointestinal tract) and Langerhans cells and macrophages (antigen-presenting cells) have been observed in the human larynx.^22^ Yamashita et al.^5^ nebulized pyokutanin blue through the nose or mouth of guinea pigs and found that mouth breathing tremendously enhanced blue staining in the arytenoid and subglottic regions, suggesting possible contact between the antigen and the laryngeal mucosa. Suzuki et al.^23^ used an environmental pollen challenge chamber to show that laryngeal symptoms in patients with cypress hay fever were markedly enhanced by pollen exposure only through the mouth, and that allergic reactions in the larynx are likely to be involved in this enhancement. These investigations indicate that the larynx is able to be locally exposed to antigens.

Chronic laryngeal allergy is distinguished into seasonal and perennial types in the 2005 diagnostic criteria.^17^ These criteria are further divided into broad and strict categories.^17^ The broad criteria are used in the daily clinical setting, whereas the strict criteria are applied in medical research. Patients with laryngeal allergy diagnosed using the strict criteria for perennial laryngeal allergy proposed in 2005 showed prominent improvements after the administration of oral histamine H1 receptor blocker (antihistamine).^24^ This suggested that the criteria were adequate for application in an actual clinical setting. Furthermore, Katada et al.^25^ investigated 159 patients with hay fever caused by birch pollen and found that the 2005 broad criteria for diagnosing seasonal laryngeal allergy could be used to distinguish laryngeal allergy from oral allergy syndrome.

In 2011, these criteria for laryngeal allergy were revised to improve the diagnostic accuracy, as shown in Tables 1, 2, 3, and 4.^17^ These are the most current criteria, and the 2019 JRS guidelines recommend the clinical use of the 2011 broad criteria for perennial laryngeal allergy by general physicians.^6^

The causative antigens might be easier to identify in patients with seasonal laryngeal allergy than those with perennial laryngeal allergy; the antigens commonly involved in seasonal laryngeal allergy include cedar, cypress, grass, or weed pollens. Imon et al.^26^ compared the antigens of patients with perennial allergic rhinitis without laryngeal symptoms with those of patients with laryngeal allergy. Both groups showed marked sensitivity to house dust and mites, but there were significantly more patients with sensitivities to moths and cockroaches in the laryngeal allergy group. Thus, these insect antigens may be specific causes of perennial laryngeal allergy.

**Figure 1:**
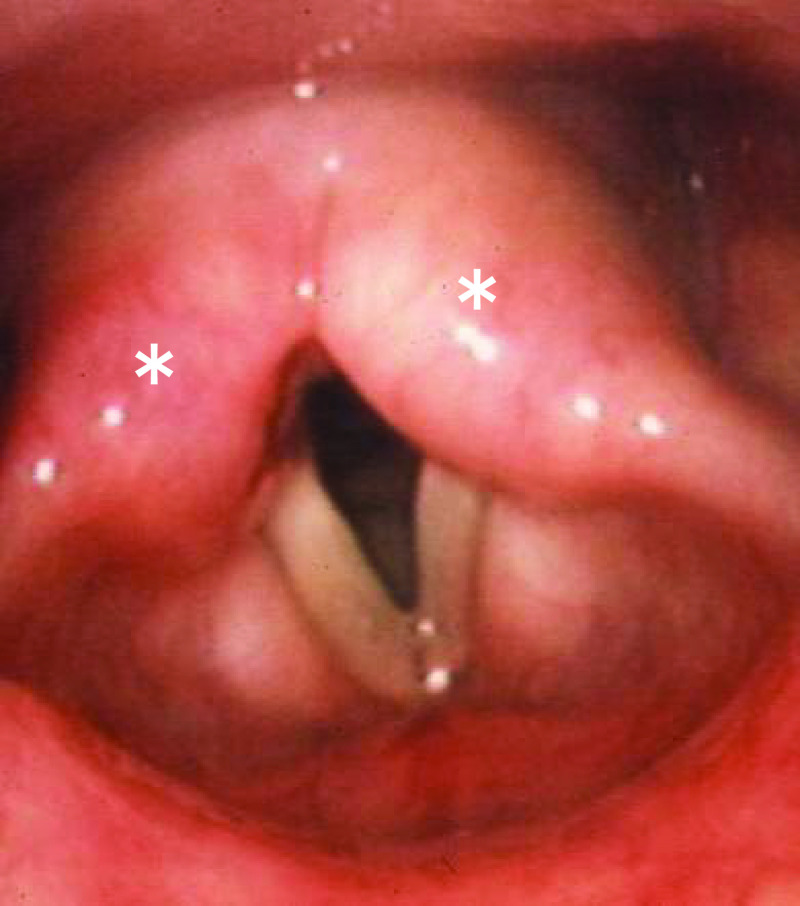
Characteristic laryngeal findings, pallor, and mild swelling of the arytenoid (*) observed in patients with chronic laryngeal allergy.

Perennial laryngeal allergy has been distinguished from other similar diseases, including CVA, AC, EB, PIC, PNDS, and GERD.^2,3^ Therefore, clinical studies that apply the 2011 strict criteria for perennial laryngeal allergy are warranted to determine the accuracy of the diagnostic criteria. Imon et al.^26^ demonstrated that the 2011 strict criteria are effective in diagnosing perennial laryngeal allergy by comparing the laryngeal findings and the efficacy of antihistamine administration in patients with allergic laryngitis versus those with non-allergic laryngitis. A pale and edematous arytenoid were typical local findings in chronic laryngeal allergy (Figure 1). Moreover, the efficacy of antihistamine administration was significantly greater in the allergic laryngitis group than in the non-allergic laryngitis group.

The use of antihistamine, which is an effective treatment for chronic laryngeal allergy, has been included in the diagnostic criteria. Imoto et al.^27^ clinically examined the efficacy of antihistamine in alleviating laryngeal symptoms in patients with cedar pollinosis resistant to leukotriene receptor agonist therapy; as antihistamine was found to be effective in managing the laryngeal symptoms of patients with cedar pollinosis, it was nominated as the specific therapy for seasonal laryngeal allergy.

Laryngeal allergy is considered to be a rare condition in children because of the small number of reported cases.^28^ Moreover, it is difficult to determine whether laryngeal allergy is the only cause of persistent cough in children, as there are several causes of chronic cough in children, such as PNDS, BA, allergic rhinitis, GERD, and psychogenic cough.

## Differential diagnoses

Laryngeal allergy has been distinguished from several diseases that cause persistent cough.^29,30^ The most common differential diagnoses for laryngeal allergy are CVA,^31^ AC,^32^ allergic bronchitis,^33^ and EB.^34^ Fundamentally, these diseases do not show apparent abnormal findings in the lungs, in contrast to tuberculosis, cancer, and fibrosis. Table 5 shows the clinical features of these differential diagnoses for laryngeal allergy.^3^ The incidences of allergic bronchitis and EB in Japan have decreased over time.

BA causes stridor, dyspnea, and persistent dry cough, whereas CVA only causes cough without stridor and dyspnea. The pathology of CVA is relatively similar to that of BA, and both conditions can only be treated by the administration of a bronchodilator and/or inhaled corticosteroid, not by the administration of oral antihistamine.^31^

It is difficult to distinguish AC from laryngeal allergy, as both conditions cause persistent dry cough, globus, atopic factors, the absence of bronchial hyperresponsiveness, and are responsive to antihistamines.^17,32^ Thus, laryngeal allergy and AC are included in the same category in the diagnostic flowchart of the 2019 JRS guidelines.

PND is a common cause of chronic cough.^35^ PND presents as a productive cough, while other causes of cough usually trigger a dry cough.^36^ Chronic sinusitis is assumed to be the most feasible reason for PND. Chronic sinusitis is also sometimes accompanied by BA; hence, it is important to determine whether patients with chronic sinusitis have a cough caused by PND, BA, or both conditions.

PIC is a complex condition that is difficult to distinguish from other similar conditions.^37^ This prolonged cough following a common cold typically demonstrates the natural course of improvement without specific treatment.^37^

GERD is an important condition that must be distinguished from laryngeal allergy.^38,39^ In Japan, the first case of persistent cough due to GERD was reported in 1992 by Fujimori et al.^40^ Cough and/or globus caused by GERD is primarily treated by the administration of a proton pump inhibitor. Surgical treatment might be recommended for some GERD patients who are resistant to proton pump inhibitors.^41^

The presence of a foreign body in the airway can cause persistent coughing.^42^ Hence, the otolaryngologist should look for a transparent foreign body in the respiratory tree on X-ray. Furthermore, computed tomography is useful in identifying foreign bodies that are not apparent on chest X-ray.^6^

Psychogenic cough is described as barking, foghorn, and brassy.^43^ Patients with this condition cough only in the daytime and sleep well at night, while patients with other types of persistent cough often experience insomnia. Psychogenic coughing usually occurs in school-aged and adolescent patients.^6^

Shimizu et al.^44^ reported that the cause of chronic cough is unknown in 7% of patients. We reported a case of chronic cough with unknown causes that was initially suspected to be caused by laryngeal allergy.^45^ In recent decades, the number of individuals in Japan with specific IgE to mites and/or pollens has increased considerably.^46^ Therefore, more attention should be paid to chronic cough and/or globus due to atopic factors.

Finally, Shimizu et al.^44^ described several adult patients with persistent cough caused by overlapping reasons such as laryngeal allergy and GERD or CVA and GERD. Consequently, clinicians should consider several causes of cough, even in adults.

## Conclusion

Recently, many clinicians have reported an increasing number of patients with persistent cough and/or globus. Studies suggest that chronic laryngeal allergy is an important cause of these symptoms. Although useful investigations into laryngeal allergy have been conducted,^1–5,21,24–28^ some questions related to this subject remain unresolved. Accurate differentiation of chronic laryngeal allergy from similar conditions is required to control the cough and/or globus and prevent the unnecessary use of medical resources. Therefore, several investigations are proposed to better understand laryngeal allergy and similar diseases, including a comparison of the accumulation of eosinophils or mast cells in the laryngeal and bronchial mucosa between patients with laryngeal allergy versus those with CVA, and large-scale double blind clinical trials evaluating the efficacy of antihistamine in patients with laryngeal allergy. More studies are required to establish the concept of laryngeal allergy.

## Figures and Tables

**Table1:** Strict diagnostic criteria for perennial laryngeal allergy (2011) (translated into English from the Japanese text in reference 17)

1.	Dry cough without wheezing for more than 8 weeks
2.	Foreign body, itching, ticklishness, and/or tingling sensation in the larynx for more than 8 weeks
3.	Atopic factors*^1^
4.	No definitive evidence of acute inflammation, infection (diphtheria, tuberculosis, or syphilis), mycosis, foreign body, or tumor in the larynx
5.	Normal pulmonary function and chest X-ray findings
6.	No findings of gastroesophageal reflux disease*^2^ or postnasal drip syndrome*^3^
7.	Complete or marked effectiveness of treatment with H1 blockers

*1. Atopic factors (at least one of the findings listed below) (1) History of allergic diseases, except for classic bronchial asthma (2) Peripheral blood eosinophilia (3) Elevated total IgE level in serum (4) Positive for allergen-specific IgE in serum (5) Positive skin reaction to allergen(s) *2. Findings of gastroesophageal reflux disease (at least one of the findings listed below) (1) Abnormal range of 24 h pH level in the esophagus (2) Abnormal esophageal fiberscopic findings (3) Abnormal findings on esophagography (4) Response to proton pump inhibitors (5) Heartburn and belching *3. Findings of postnasal drip syndrome (at least one of the findings listed below) (1) Postnasal drip (2) Positive findings on visual inspection (3) Positive findings on nasal fiberscopic examination

**Table2:** Broad diagnostic criteria for perennial laryngeal allergy (2011) (translated into English from the Japanese text in reference 17)

1.	Dry cough without wheezing for more than 3 weeks
2.	Foreign body, itching, ticklish, and/or tingling sensation in the larynx for more than 3 weeks
3.	Atopic factors*^1^
4.	No definitive evidence of acute inflammation, infection (diphtheria, tuberculosis, or syphilis), mycosis, foreign body, or tumor in the larynx
5.	Moderate effectiveness of treatment with H1 blockers

*1. Atopic factors (at least one of the findings listed below) (1) History of allergic diseases, except for classic bronchial asthma (2) Peripheral blood eosinophilia (3) Elevated total IgE level in serum (4) Positive for allergen-specific IgE in serum (5) Positive skin reaction to allergen(s)

**Table3:** Strict diagnostic criteria for seasonal laryngeal allergy (2011) (translated into English from the Japanese text in reference 17)

1.	Dry cough without wheezing during the pollination season
2.	Foreign body, itching, ticklish, and/or tingling sensation in the larynx during the pollination season
3.	Proof of type I allergy to causal pollen*^1^
4.	No definitive evidence of acute inflammation, infection (diphtheria, tuberculosis, or syphilis), mycosis, foreign body, or tumor in the larynx
5.	Normal pulmonary function and chest X-ray findings
6.	Absence of gastroesophageal reflux disease*^2^ and postnasal drip syndrome*^3^
	7. Complete or marked effectiveness of treatment with H1 blockers

*1. Proof of type I allergy to pollen (at least one of the findings listed below) (1) Positive skin reaction to causal pollen (2) Pollen-specific IgE detected in serum *2. Findings of gastroesophageal reflux disease (at least one of the findings listed below) (1) Abnormal range of 24 h pH level in the esophagus (2) Abnormal esophageal fiberscopic findings (3) Abnormal findings on esophagography (4) Response to proton pump inhibitors (5) Heartburn and belching *3. Findings of postnasal drip syndrome (at least one of the findings listed below) (1) Postnasal drip (2) Positive findings on visual inspection (3) Positive findings on nasal fiberscopic examination

**Table4:** Broad diagnostic criteria for seasonal laryngeal allergy (2011) (translated into English from the Japanese text in reference 17)

1.	Dry cough without wheezing during the pollination season
2.	Foreign body, itching, ticklish, and tingling sensation in the larynx during the pollination season
3.	Proof of type I allergy to causal pollen*^1^
4.	No definitive evidence of acute inflammation, specific infection (diphtheria, tuberculosis, or syphilis), mycosis, foreign body, or tumor in the larynx
6.	Moderate effectiveness of treatment with H1 blockers

*1. Proof of type I allergy to pollen (at least one of the findings listed below) (1) Positive skin reaction to causal pollen (2) Pollen-specific IgE in serum

**Table5:** Clinical features of the differential diagnoses for laryngeal allergy (modified from reference 33)

	CVA	Atopic cough	Allergic bronchitis	EB	Laryngeal allergy
Symptom	Dry cough	Dry cough	Dry cough	Dry cough	Dry cough
Duration	More than 1 month	More than 8 weeks	Persistent	Chronic	More than 8 weeks
Atopic factors	+	+	+	+	+
Methacholine sensitivity	+	−	−	−	−
Bronchodilator	Effective	Ineffective	Ineffective	Ineffective	Ineffective
Cough suppressant	Ineffective	Ineffective	Ineffective	Ineffective	Ineffective
Steroid	Effective	Effective	Effective	Effective	Unknown
Antihistamine	Ineffective	Effective	Ineffective	Unknown	Effective
Outcome	Progression to asthma	No progression to asthma	Unknown	Unknown	Unknown

CVA: cough variant asthma, EB: eosinophilic bronchitis without asthma
